# Prevalence of Depression and Suicidal Ideation and Associated Risk Factors in Adolescents Receiving Care and Treatment for Hiv/Aids at a Tertiary Health Facility in Kilimanjaro Region, Tanzania

**DOI:** 10.21203/rs.3.rs-2534893/v1

**Published:** 2023-02-28

**Authors:** Editruda Gamassa, Ester Steven, Rachel Mtei, Sylvia Kaaya

**Affiliations:** Kilimanjaro Christian Medical Centre; Muhimbili University of Health and Allied Sciences; Muhimbili University of Health and Allied Sciences; Muhimbili University of Health and Allied Sciences

**Keywords:** depression, suicidal ideation, adolescents, HIV/AIDS

## Abstract

**Background:**

The HIV/AIDS pandemic is a significant health concern worldwide since the first case emerged in the early 1980’s. Most of the HIV disease burden is in Sub Saharan Africa (SSA). Depression and suicidal ideation have been found to be higher among people living with HIV/AIDS (PLWHA) than persons not infected with HIV. Adolescents living with HIV/AIDS (ALWH) are more at risk of suffering from depression and suicidal ideation than their unaffected peers. Factors such as social demographics, poor social support and HIV related stigma have been found to be significantly associated with depression and suicidal ideation among adolescents living with HIV/AIDS. Moreover, depression and suicidal ideation may lead to poor ART adherence, lower viral load suppression and development of opportunistic infections. Few studies have evaluated the mental health of ALWH in SSA. However, these studies do not provide information on the magnitude of suicidality among this population subgroup despite them being at high risk.

**Objective:**

The aim of the study is to determine the prevalence of depression and suicidal ideation and explore associated risk factors in adolescents receiving care and treatment for HIV/AIDS at a tertiary health facility in Kilimanjaro Region, Tanzania.

**Materials and methods:**

A hospital-based cross-sectional analytical study using quantitative approach was conducted. Adolescents receiving care and treatment for HIV/AIDS in a youth clinic at Kilimanjaro Christian Medical Centre (KCMC) were sampled. Data collection on depression was assessed using Patient Health Questionnaire for Adolescents (PHQ-A). A semi-structured questionnaire captured the socio-demographic and clinical information characteristics of the participants, also included a short version of the HIV Stigma Scale measured HIV-related stigma, a social support measure (Multidimensional Perceived Social Support Scale (MSPSS)) and a locally developed Hope Scale assessed hopefulness. Captured data were analyzed using SPSS version 23; Frequency distributions described the participants’ sociodemographic characteristics. Chi-Square test established the univariate association between the independent and the dependent variables. While analysis to identify independent risk factors of suicidality and depression was used a multivariate logistic regression model. Associated risk factors and the strengths of association are summarized using odds ratios (ORs) and 95% confidence intervals. Ethical clearance was obtained from MUHAS Senate Research and Publications Committeeand permission sought from the administration of KCMC.

**Results:**

A total 170 adolescents were studied, 96 (56.5%) were females while 74 (43.5%) were males. Mean age (standard deviation) of participants was 15 (2.2) years. The prevalence of depression was 15.9% and that of suicidal ideation was 31.2%. Based on univariate analysis relatively high levels of HIV-related stigma and lower levels of hope were significantly associated with depression and suicidal ideation. From multivariable analyses adolescents with low levels of hope were 9.2 times more likely to develop depression compared to those with high levels of hope (OR, 9.21, 95% CI: 2.16-39.32).

Participants who experienced high levels of HIV-related stigma were 2.7 times more likely to have suicidal ideation compared to those with lower levels (OR, 2.7, 95% CI: 1.01-7.37). Furthermore, participants with low levels of hope were also 3.8 times more likely to have suicidal ideation compared to those with high levels (OR, 3.84, 95% CI: 1.50-9.84).

**Conclusion and recommendation:**

This study reveals depression and suicidal ideation among adolescents living with HIV to be 16% and 31% respectively. High levels of stigma and low hope were identified as risk factors.

Further studies need to be done to evaluate the mental health of adolescents living with HIV and integration of mental health services in the clinic providing services for these adolescents.

## Introduction

Human Immunodeficiency Virus (HIV) and its related disorders constitute a global health threat and continue to pose a public health challenge. It is estimated that 37.9 million people globally, are living with HIV. Among this population, 1.6million are adolescents between the ages of 10-19 years. Sub-Saharan Africa (SSA) accounts for the majority of world’s population of HIV infected individuals, whereby 89% of the adolescents with HIV reside in SSA (1). The prevalence of HIV infection in Tanzania is 5.7%. Approximately 81,000 adolescents were living with HIV in Tanzania in 2016 (2).

HIV infection has an adverse effect on the mental health of an individual due to the direct effect to the brain causing neurocognitive changes as well as the indirect effects of the psychological distress of living with a chronic illness that impacts self-care practices (3). Depression is one of the common mental disorders affecting people living with HIV/AIDS (PLWHA) (4). Depression in children to adolescents is characterized by persistent, impairing sadness, anhedonia and irritability; and mood changes that are relatively unresponsive to pleasurable activities and interactions or attention from other people (Rutter’s Child and Adolescent Psychiatry Sixth Edition).. Studies done in SSA show prevalence of prevalence of depressive symptoms among adolescents living with HIV/AIDS to range between 7.6% to 48.8% (5-7)(5-8). The odds of depressive disorder is almost two-fold higher among adolescents living with HIV compared to their HIV-unaffected peers (9)

Suicide ideation are thoughts of engaging oneself in acts or behaviors intended to end one’s life, including wishes to kill oneself and may lead to making plans of when, where and how to carry out the act. (10). The prevalence of suicidal ideation is higher in adolescents compared to the general population. A Canadian study showed the prevalence of suicidality (suicidal ideation and attempt) in adolescents to be 13.5% while a study done in 59 low- and middle-income countries showed the prevalence of suicidal ideation to be 16.9% with the highest pooled prevalence being in Africa (11,12). Adolescents living with HIV/AIDS (ALWHA) have increased risk of suicidal ideation compared to the unaffected population, this is due to the psychological distress they endure as a result of biological effect of HIV on the brain or/and the stress of living with a chronic illness that may impair personality development and function (13,14). The prevalence of suicidal ideation among adolescents living with HIV/AIDS in devolved countries ranges from 9% to 29% (15-17), while in SSA the prevalence ranges from 18% to 33% (6,18-20). There are several social demographic and psychosocial factors associated with depression and suicidal ideation among ALWHA (21). Female sex is highly associated with suffering from depression or suicidal ideation among adolescents this may be due to the females tendency of internalizing stressful events (19,22). Being an orphan increases vulnerability for suffering mental disorder, that is thought to be mediated by psychological distress and impaired social support (23,24). High levels of social support contribute to the overall stress buffering hence moderating the effects of stigma in adolescents living with HIV (25). HIV positive adolescents with low and moderate social support are twice likely to develop depressive symptoms compared to those with strong social support (5). Family and peer led intervention enhances support among adolescents living with HIV and thus may be useful in the prevention and management of depression. (26). Due to the negative effects of HIV related stigma, PLWHV are at high risk of suffering from mental health disorders such as depression and anxiety (21,27). Several studies have shown that adolescents facing internalized HIV stigma have high rate of depression and suicidal ideation (22,25,28). Hope is associated with HIV as a psychological factor, it may affect the treatment and care of individuals living with HIV and quality of life (29). Feeling of hopelessness was found to be associated with depression and increased risk of suicidal behavior (13).

These factors affect adolescents biologically, psychologically and socially; and may compromise young persons’ coping ability and risk to develop mental disorders. Mental disorder challenge adaptive coping and illness self-management skills that may worsen HIV/AIDS progression and health-related quality of life. Assessment of such factors will offer pathways for intervention, enhance adherence to treatment and medication and improve quality of life.

Limited studies have been done in Tanzania to estimate the prevalence of depression and suicidal ideation among adolescents living with HIV. Therefore, this study aimed to estimating the prevalence of depression and suicidal ideation and associated factors among adolescents living with HIV and receiving care at KCMC.

## Methodology

### Study design

This was a hospital-based cross-sectional study that utilized quantitative methods, conducted from February to April 2021.

#### Study area

The study was conducted at KCMC, a tertiary referral hospital in Tanzania located on the foothills of Mt Kilimanjaro and serving more than 15 million people in Northern Tanzania. KCMC has three dedicated HIV clinics, namely Care and Treatment Clinic (CTC), Child-Centered Family Care Clinic (CCFCC), and the Infectious Disease Clinic (IDC). Approximately 800 patients receive care and treatment in these clinics. This study was conducted at the CCFCC, a HIV-youth-focused clinic that is specialized for providing care and treatment to adolescents and youths living with HIV/AIDS. The clinic is held once a month on the last Saturday of the month and attends to more than 100 adolescents/youth.

#### Study population

The study population included the adolescents receiving care at the CCFCC. Data was collected from adolescents who provided assent and also consent was obtained from parents.

### Sample size calculation

The researcher used a prevalence rate from a previous study and used the formula to calculate the sample size. The prevalence from that study is 12.1% (7). Therefore, total projected sample size was 179.

### Sampling and Data Collection Procedure

Consecutive sampling technique was used to recruit the participants. The tools used, (Sociodemographic Questionnaire, PHQ 9-A, HIV stigma scale, Multidimensional Perceived Social Support Scale [MSPSS] and Hope scale) were compiled into one research document.

### Sample Selection Criteria

#### Inclusion criteria

1. Adolescents living with HIV aged between 10–19 years receiving care at KCMC;
2. Consented to participate in the study as follows;
  - Adolescents aged 10 to 17 who gave assent to participate in the study and followed by their parental/guardian’s consent,
  - Adolescents aged 18 and 19 able to provide consent.

## Exclusion criteria

1. Adolescents with serious mental illness such as severe intellectual disability, acute/chronic confusion state, agitation/aggressiveness hence unable to give consent or reliable information due to their mental status.
2. Adolescents who were physically unable to participate.

### Variables

#### The independent variables

1. Sociodemographic factors
2. HIV-related stigma
3. Social support
4. Clinical factors

#### The dependent variables

Depression

Suicidal ideation

## Results

A total of 170 adolescents were eligible, assented and their parents/guardians consented for participation in the study. Data from 170 (100%) participants who participated in the study were complete and used in those analyses. Female adolescents constituted 56.5% (96) of all adolescents. The age range was between 10–19 with the mean age of 15.1 years. Most adolescents were still enrolled to school, 94.1% (160), of which 61.2% (104) had secondary school education and 29.4% (29) primary education (Table 1).

Of all the participants, 25.9% (44) experienced high level of HIV-related stigma whereby the rest equally experienced low or moderate levels 37.1% (63). The majority of adolescents had moderate perceived social support 54.1% (92) followed by high-perceived social support 37.1%( 63) while only 8.8%(15) had low levels of perceived social support. Hope was another psychological component assessed whereby moderate levels of hope were observed in 34.7% (59) of the participants followed by high level of hope of which 33.5% (57) and 31.8% (54) had low level of hope (Table 1).

**Figure 1:**
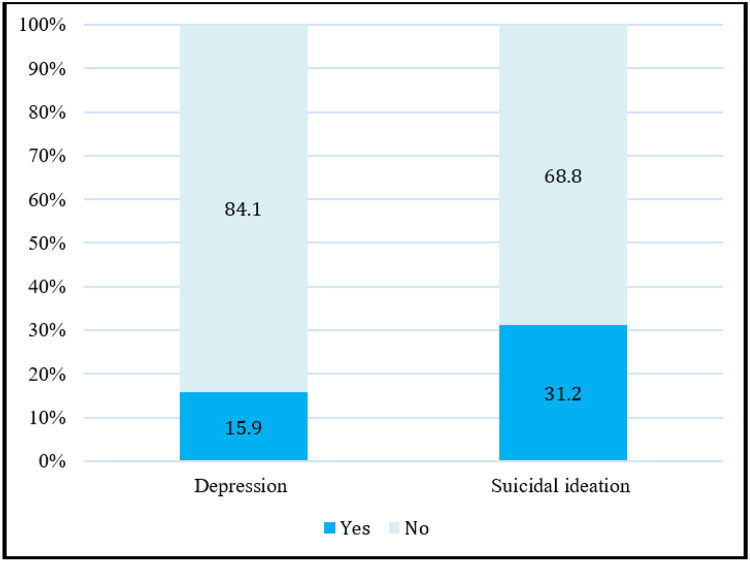
Prevalence of Depression and Suicidal Ideation Among Adolescents receiving care and treatment for HIV/AIDS at KCMC.

According to the PHQ-A scores; and using 5 as the cut-off point for probable depression. Of which, 15.9% (27) of participants had depression, while the rest 84.1% (143) did not have depression. Over a course of one month, 31.2% (53) of the participants had thoughts about ending their life and 14.7% (25) had lifetime attempted suicide (Fig. 1).

In bivariate analysis, hope was the only psychosocial factor associated with depression and suicidal ideation.

Multivariate analysis showed that, adolescents with low levels of hope were 9.2 times and 3.8 times more likely to develop depression and suicidal ideation compared to those with high level of hope (OR, 9.21, 95% CI:2.16–39.32) (OR, 3.84, 95% CI: 1.50–9.84) (Table 2 &3). Furthermore, participants who experienced high level of HIV-related stigmas were 2.7 times more likely to have suicidal ideations compared to those with low level (OR, 2.7, 95% CI: 1.01–7.37) (Table 3).

## Discussion

The broad objective of this study was to assess the prevalence of depression and suicidal ideation and its associated factors among adolescents living with HIV. Specifically, the current study examined the effect of sociodemographic characteristics, HIV-related stigma, perceived social support and hope on adolescents with depression and suicidal ideation.

Findings of this study revealed the prevalence of depression among adolescents living with HIV to be 15.9% by the cut-off point of 5 on the PHQ-A. It almost similar to a Ugandan study in 2018 whereby among 224 adolescents living with HIV, 16% had major depressive disorder as classified using MINI kid (28). Furthermore, the finding is slightly higher compared to a study done in Moshi, Tanzania in 2014 to assess mental health difficulties of adolescents living with HIV, of which prevalence of depressive symptoms was 12.1% by using PHQ-9 with >10 as a cutoff point (7) . However, this finding is lower compared to other studies done in SSA, a study from Kenya showed that 17.8% of HIV-infected children and adolescents between 6- 18 years had major depressive disorder by using Mini International Neuropsychiatric Interview for Children and Adolescents (MINI kid) (Kamau et al., 2014.). Another study from Ethiopia showed that 35.5% of HIV positive youths aged 15- 24 years had depressive symptoms as screened by using Beck Depression Inventory II (5).

In comparison with developed countries, studies done in USA and Thailand showed the prevalence of depression to be higher than this finding. In the USA, study among youths aged 11- 25 years, showed the prevalence of depression to be 24% and in Thailand it was found to be 27.8% (16,17). Patient Health Questionnaire (PHQ-9) and Thai Children’s Depression Inventory (CDI) were used to screen for depression in these studies.

The difference could be due to long duration between the two studies, and psychosocial issues related to the time of study i.e. this study was done during COVID 19 pandemic, also (7) used an adult screening tool with higher cut off points. The Kenyan study show a higher prevalence compared to this study, this is because (Kamau et al., 2014) used a diagnostic tool for DSM-IV criteria to diagnose depression. The variations in prevalence between countries/ continents could be due to several factors such as variation tools; some used diagnostic tools while others screening tools with valid cut off scores.

The study found the prevalence of suicidal ideation to be 31.2% using the PHQ-A tool. This finding is higher compared to most of the studies done in SSA. Study from Ethiopia showed that 27.1% of youths living with HIV had suicidal ideation (18). Another study in Nigeria showed the prevalence of current and lifetime suicidal ideation to be 14.9% and 33.3% respectively (19). In Kenya, a study done in 2016 found 18% of the adolescents had suicidal risk but not attempts or plans with variations on age, whereby the older adolescents had higher rate of suicidal risk than the younger adolescents (Kamau et al., 2014). Another study from South Africa showed only 8% of adolescents living with HIV had suicidal thoughts (25). The prevalence in this study is higher due to the nature of PHQ-A tool adapted for adolescent populations with a specific focus on suicidality, which is common in this age group.

The prevalence of suicide attempt is 14.7%, this is slightly lower compared to an Ethiopian study which showed prevalence to be 16.9% (18). However, the finding is higher compared to a study in South Africa which show prevalence of suicide attempt to be 4%, assessed by the Mini International Psychiatric Interview for Children and Adolescents Suicidality and self harm subscale (25). In a Kenyan study by (Kamau et al., 2014) no adolescent reported any suicide attempt.

Findings of this study showed that, there were no sociodemographic factors that were associated with depression and suicidal ideation among adolescents living with HIV. This varies from other studies done in SSA whereby, different sociodemographic factors had influences on diagnosis of depression or suicidal thoughts among adolescents living with HIV. Studies from Kenya and Ethiopia showed that adolescents/ youths in the older age group i.e from 15 – 24 years had increased risk of depression and suicidal thoughts (5,6). Sex has also been found to be highly associated with depression and suicidal ideation among adolescents living with HIV (19).

High social support is believed to be a one of the stress buffers in moderating psychosocial factors predisposing an individual to depression or suicidal ideation. However, results from this study show no significant association between social support and depression or suicidal ideation among adolescents living with HIV. Several studies have shown that adolescents with high social support were less likely to have depressive symptoms or suicidal thoughts (5,8,18). Overall, it is important to have family and peer led interventions so as to enhance support among adolescents living with HIV and thus useful in prevention and management of depression and suicidal ideation.

Findings of this study have shown that adolescents experiencing HIV-related stigma had increased risk of having suicidal ideation. This is similar to study done among South African adolescents whereby, those who had higher HIV related stigma were more likely to have depression and suicidal ideation (25). Moreover, Studies done in Ethiopia showed that HIV-related stigma was significantly associated with depressive symptoms and suicidal ideation among youths infected with HIV. Also in Rwanda a study done to compare mental health of adolescents infected with HIV and those unaffected showed that HIV-infected adolescents faced higher rate of stigma than the unaffected group and hence increased their risk of depression and other mental health challenges (5,27)

Hope can be described as believing life to be worth living at the present and in the future (30). This study has found that adolescents with low hope were 9 times likely to have depression and 3.8 times likely to have suicidal ideations. This finding is similar to studies done SSA among PLWH showed that feeling of hopelessness predisposed an individual to mental disorders such as depression, anxiety and suicidality (29,31). The tool used to assess hope is new and a study to set the cutoff points on the hope scale used for this population is of paramount importance, as it has not yet been done.

## Conclusion, Recommendations And Limitations

This study aimed to assess prevalence of depression and suicidal ideation and associated factors among adolescents living with HIV receiving care at KCMC. The findings revealed the prevalence of depression and suicidal ideation to be 15.9% and 32. 5% respectively. Psychosocial factors such as stigma and hope have been found to be associated with depression and suicidal ideation among this population.

### Recommendations

From the findings of this study, we recommend the following:

- Regular screening and early intervention for depression in all patients attending treatments should be done.
- We suggest implementation research, adapting mental health intervention for adolescents living with HIV, who have depression and other mental health concerns.

#### Study strengths

- It is one of the few studies that has explored mental health concerns in AWHIV.

#### Study limitations

- Recall bias could have interfered with the results in answering time-framed questions such as two weeks for depression.
- The following tools have not been validated in Tanzania.
  - Patient Health Questionnaire 9 Adolescent version (PHQ-A)
  - HIV Stigma Scale
  - Multidimensional Scale for Perceived Social Support Scale (MSPS)

## Figures and Tables

**Table 1:** Sociodemographic and psychosocial characteristics of adolescents receiving care and treatment for HIV/AIDS at KCMC

Item	N	%
**Age**	**67**	**39.4**
**10–14**	**103**	**60.6**
**15–19**		
**Sex**	**74**	**43.5**
**Male**	**96**	**56.5**
**Female**		
**Level of education**	1	0.6
Never enrolled	50	29.4
Primary school	104	61.2
Secondary school	15	8.8
College/university		
**Orphan status**	80	47.1
No	25	14.7
Both parents	65	38.2
One parent		
**HIV-related stigma**	63	37.1
Low	63	37.1
Moderate	44	25.9
High		
**Social support**	15	8.8
Low	92	54.1
Moderate	63	37.1
High		
**Age**	**67**	**39.4**
**10–14**	**103**	**60.6**
**15–19**		
**Sex**	**74**	**43.5**
**Male**	**96**	**56.5**
**Female**		
**Hopefulness**	54	31.8
Low	59	34.7
Moderate	57	33.5
High		

**Table 2:** Logistic regressions of independent factors of depression among adolescents receiving care and treatment at KCMC

Variable	OR95%CI	p-value	AOR95%CI	P-value
***Age***				
10–14	Ref		Ref	
15–19	1.127(0.482,2.636)	0.783	0.754(0.274,2.071)	0.583
Sex				
Male	Ref		Ref	
Female	1.145(0.497,2.642)	0.750	1.190(0.47,3.024)	0.715
Orphan status				
Not orphan	Ref		Ref	
Both parents	2.250(0.663,7.638)	0.193	2.085(0.53,8.28)	0.297
One parent	2.471(0.965,6.323)	0.059	1.968(0.68,5.671)	0.210
HIV-related stigma				
Low	Ref		Ref	
Moderate	1.333(0.464,3.834)	0.593	0.780(0.232,2.623)	0.688
High	2.667(0.942,7.550)	0.065	1.640(0.502,5.356)	0.413
Social support				
Low	2.000(0.451,8.868)	0.362	0.933(0.170,5.122)	0.936
Moderate	1.813(0.704,4.669)	0.217	0.922(0.303,2.806)	0.887
High	Ref		Ref	
Hope				
Low	9.771(2.690,35.490)	0.001	9.212(2.158,39.318)	0.003
Moderate	1.667(0.379,7.323)	0.499	1.753(0.375,8.201)	0.476
High	Ref		Ref	

**Table 3:** Logistic regression of independent factors of suicidal ideation among adolescents receiving care and treatment at KCMC

Variable	OR95%CI	P-value	AOR95%CI	P-value
***Age***				
10–14	Ref		Ref	
15–19	1.401(0.712,2.757)	0.329	1.110(0.497,2.479)	0.799
Sex				
Male	Ref		Ref	
Female	0.722(0.376,1.387)	0.329	0.607(0.286,1.289)	0.193
Orphan status				
Not orphan	Ref		Ref	
Both parents	2.000(0.776,5.155)	0.151	1.898(0.642,5.610)	0.246
One parent	1.643(0.802,3.366)	0.175	1.135(0.491,2.624)	0.768
HIV-related stigma				
Low	Ref		Ref	
Moderate	2.125(0.937,4.817)	0.071	1.675(0.673,4.168)	0.267
High	3.542(1.492,8.408)	0.004	2.732(1.012,7.372)	0.047
Social support				
Low	1.273(0.351,4.620)	0.714	0.529(0.116,2.408)	0.410
Moderate	2.149(1.038,4.451)	0.039	1.500(0.649,3.465)	0.343
High	Ref		Ref	
Hope				
Low	5.054(2.194,11.644)	0.000	3.837(1.495,9.844)	0.005
Moderate	0.765(0.301,1.943)	0.574	0.579(0.215,1.562)	0.281
High	Ref		Ref	
